# Association of *OXTR*, *AVPR1a*, *LNPEP*, and *CD38* Genes’ Expression with the Clinical Presentation of Autism Spectrum Disorder

**DOI:** 10.3390/cimb45100527

**Published:** 2023-10-16

**Authors:** Krzysztof Maria Wilczyński, Aleksandra Auguściak-Duma, Aleksandra Stasik, Lena Cichoń, Alicja Kawalec, Małgorzata Janas-Kozik

**Affiliations:** 1 Department of Developmental Age Psychiatry and Psychotherapy, Medical University of Silesia, 40-061 Katowice, Poland; 2 John Paul II Children’s and Family Health Center in Sosnowiec sp. z o.o., Gabrieli Zapolskiej 3, 41-218 Sosnowiec, Poland; 3 Department of Molecular Biology and Genetics, Medical University of Silesia, 40-061 Katowice, Poland

**Keywords:** oxytocin, expression, autism

## Abstract

Autism spectrum disorder (ASD) is a complex neurodevelopmental disorder that affects social interactions, communication, and behavior. Although the predominant genetic predisposition to ASD seems beyond doubt, its exact nature remains unclear. In the context of social cognition disorders and the basis of ASD, the oxytocinergic and vasopresynergic systems arouse great interest among researchers. The aim of the present study was to analyze gene expression levels for oxytocin and vasopressin receptors, as well as CD38 protein and oxytocinase, in the context of the clinical picture of autism spectrum disorders. The study included 90 people, of whom 63 were diagnosed with ASD based on anamnesis, mental status testing, and the ADOS-2 protocol. The results obtained in the presented study indicate that the balance between the levels of expression of the CD38 gene and the oxytocinase gene plays a key role in the risk and clinical presentation of ASD. In a hypothetical scenario, an imbalance in the expression of CD38 and LNPEP could potentially lead to alterations in the concentrations of oxytocin and vasopressin. At the same time, the most frequently studied genes—AVPR1a and OXTR—seem to be at best of marginal importance for the risk of ASD.

## 1. Introduction

Establishing and maintaining relationships is a key aspect of human evolution that significantly impacts mental health and the overall quality of life. Autism spectrum disorder (ASD) is a complex neurodevelopmental disorder that affects social interactions, communication, and behavior. It is characterized by deficits in social cognition, including theory of mind and empathy, as well as difficulties in interpreting non-verbal communication. These challenges can lead to difficulties in maintaining mutual interactions with others and hinder the ability to share interests and understand metaphor [1]. Although the predominant genetic predisposition to ASD seems beyond doubt, its exact nature remains unclear. In general, it is assumed that, in most people, the risk of ASD may be due to frequent variants (i.e., polymorphisms) of negligible importance, which, as the number of proautistic alleles increases, will determine its occurrence. In the minority of cases (5–10%), rare variants (i.e., copy–number variations) of very high importance are present that may determine the occurrence of ASD individually. In the first case, it is possible that specific genes are responsible not so much for the entire clinical picture of ASD but rather for some of its symptoms [2]. Such a hypothesis would agree with the concept of the autistic continuum and explain the occurrence of autistic traits in the general population, regardless of the occurrence of a clinical diagnosis.

Understanding the neurochemical basis of communication disorders in ASD is of great interest to researchers, especially in the context of efforts to develop drugs that can alleviate their symptoms. Oxytocin (OXT) and vasopressin (AVP) are two neurohormones that have been the focus of extensive research in this area due to their comprehensive effects on human functioning [3]. These nonapeptides are characterized by remarkable evolutionary conservatism, and they or their analogues are observed among both vertebrates and invertebrates. Furthermore, in all species in which they are detected within the Eumetaoza phylum, they possess the following three essential characteristics [4]:

1. They are secreted within the nervous system;
2. Their function is modulated by sex hormones and differs between females and males;
3. They are crucial for social/reproductive behavior.

For this reason, in recent years, several publications have appeared in the literature trying to link their concentrations with the clinical picture and the risk of ASD in humans, but the results obtained do not allow for unambiguous conclusions [5]. However, their function depends not only on their concentration, i.e., in blood or cerebrospinal fluid, but also, for example, on their receptors (oxytocin receptor (OXTR) and vasopressin 1A receptor (AVPR1a)) or enzymes responsible for metabolism, i.e., the CD38 protein is responsible for the secretion of both these neuropeptides, and leucil/cystylin aminopeptidase (LNPEP) is responsible for their breakdown [6,7,8]. Disruption of the structure or expression of the genes encoding these proteins may have an effect similar to a deficiency of OXT or AVP, respectively. For this reason, in recent years, polymorphisms within their genes have aroused great interest among researchers. In the case of polymorphisms within *OXTR* and *AVPR1a*, which are the most thoroughly researched ones, the results available in the literature are contradictory [9]. However, the majority of authors indicate the existence of a relationship between their genotype and the clinical picture of social deficits in both neuroatypical and neurotypical populations. In terms of the *CD38* gene, there are currently isolated studies in the literature analyzing its relationship with the clinical picture of ASD. One is by Munesue et al., 2010 [10], which showed a significant association between two out of ten polymorphisms studied and the diagnosis of ASD, and a second one is by Lerer et al., 2010 [11], where a genotype associated with lower expression of this protein was shown to correlate with the risk of ASD in people with intellectual disabilities. Additionally, Thanseem et al. (2012) [12] showed an increase in *CD38* gene expression levels in brain tissues in people with ASD. In the case of *LNPEP*, to our knowledge, at the moment, there are no studies available in the literature analyzing its relationship with ASD, but the possible role that this protein plays in regulating AVP and OXT levels makes it an interesting object of research [6].

The main disadvantage of the literature published so far is the focus on single nucleotide polymorphisms themselves. Only individual studies, and only for the *CD38* and *OXTR* genes, analyze the levels of expression of these genes and their relationship to the severity and risk of developing ASD. This is important because virtually all polymorphisms that show associations with ASD or social behavior disorders are found in gene introns or in non-coding areas. Therefore, they do not affect the structure of proteins but rather their level of expression through incorrect interactions with promoters, enhancers, silencers, or insulators that are found in these areas. Moreover, a polymorphism analysis alone does not take into account the impact of epigenetic modifications. For this reason, the analysis of the expression levels of the analyzed genes seems to be a much more reliable method than research focusing only on the analysis of polymorphisms.

The aim of the present study was to assess the level of expression of genes associated with the function of the oxytocinergic and vasopresynergic systems and to relate it to elements of the clinical picture of ASD in the adolescent population.

## 2. Materials and Methods

### 2.1. Study Participants

The research was conducted as part of the Department of Psychiatry and Developmental Age Psychotherapy of the Medical University of Silesia in Katowice at the John Paul II Children’s and Family Health Center in Sosnowiec Sp.zo.o. in cooperation with the Department of Molecular Biology of the Medical University of Silesia in Katowice. Participants were recruited in the Silesian Voivodeship in Poland. Informed consent to participate in the study was obtained after information was provided from both parents and participants.

The criteria for inclusion in the study group included confirmation of the diagnosis of autism spectrum disorder using the ADOS-2 protocol. The exclusion criteria included the following: (1) concomitant diagnosis of another psychiatric disorder; (2) age under 12 and over 19 years; (3) concomitant intellectual disability; (4) epilepsy; (5) known genetic, neurometabolic, etc., background of the observed symptoms (e.g., fragile X syndrome)—the so-called “autism plus”; (6) poor somatic status (i.e., high fever, etc.); (7) diagnosis of serious liver, kidney, or heart disease; and (8) hypothyroidism.

The data collected in the study were pseudonymized. The study included 90 subjects with an average age of 14.59 years (95%CI: 13.98–15.2) and an average BINET IQ score of 100 (95%CI: 96.9–103.3). Participants were divided into two groups based on a confirmed or excluded diagnosis of autism spectrum disorder based on the DSM-5 and ICD-10 criteria, followed by the ADOS-2 protocol. The neurotypical group (control) included 27 participants with a mean age of 16.5 years (95%CI: 15.31–17.6), a mean IQ of 97 points (95%CI: 89–104), and a mean ADOS-2 comparative score of 1.69 points (95%CI: 1.10–2.28 points). The neuroatypical group (study) included 63 subjects with an average age of 13.9 years (95%CI: 13.2–14.5), an IQ of 101.2 (95%CI: 97.5–104.9), and an ADOS-2 comparative score of 6.54 (95%CI: 6–7). In the whole group, women accounted for 30% of the population (n = 27), with 34.9% of the neuroatypical group (n = 22) and 18.5% of the neurotypical group (n = 5). The observed difference in age was statistically significant in the Mann–Whitney U test, as was the difference between the ADOS-2 scores. The difference in gender distribution did not gain statistical significance in the χ^2^ test.

### 2.2. Psychometric Analysis

All participants were tested using the ADOS-2 protocol using modules corresponding to the age and language level of the participants. The ADOS-2 protocol is a tool that includes a set of attempts to provoke the subject into specific social behaviors. Based on the observations, the diagnostician evaluates the subject’s behavior in five categories: language and communication, reciprocity in social interactions, play/imagination, stereotypical behavior and rigid interests, and other abnormal behaviors. The result of the study is to obtain a quantitative result on two scales (social affect and restricted and repetitive behaviors) and a total result allowing for the determination of the degree of severity of symptoms associated with the autism spectrum. Further analyses focused primarily on the comparative score (ADOS:WP), the social affect domain (ADOS:SA), and the restricted and repetitive patterns of behavior and interests (ADOS:RRB). In addition, the study utilized the following:

1. The “Reading mind in the eyes” (RMiE) test—this is a tool designed by Baron-Cohen, which in the version applied in the presented study consists of 28 images presenting eye slices surrounded by four definitions of mental state, from which the subject must choose one corresponding to the presented figure. The test is performed without a time limit [13,14].
2. The “Autism Quotient” (AQ) test [15] is a questionnaire constructed in 2001 to assess the likelihood of a diagnosis of autism spectrum disorder. It consists of 50 questions with answers based on a 4-point Likert scale.
3. The “Empathy Quotient” (EQ) test is a tool designed by Baron-Cohen (translated by Jankowiak-Siuda et al., 2017), which allows for the determination of individual differences in the ability to empathize. The presented work uses the version of Wakabayashi et al. containing 22 test items [16].
4. The Stanford Binet Intelligence Scale, Fifth Edition, is a test that assesses intelligence and cognitive abilities. It is suitable for people aged 2–85 years. The full IQ scale consists of ten subscales. In our study, we used an abbreviated IQ scale consisting of two tests: series/matrices and dictionary [17].

### 2.3. Consent of the Bioethics Committee and Source of Funding

The study was conducted with the consent of the Bioethics Committee of SUM, as issued by resolution no. CDF/0022/KB1/123/18/19 on 8 January 2019.

The costs of the study were covered by the funds of the Medical University of Silesia under statutory employment contracts (no. PCN-2-067/K/0/K and PCN-2-102/K1/K).

### 2.4. Molecular Analysis

RNA expression analysis. The mRNA was extracted from peripheral blood collected into Pax tubes using the PaxGene Blood RNA Kit (Qiagen, Germany) in order to preserve RNA integrity, following the manufacturer’s guidelines. Qualitative and quantitative analyses of RNA were carried out on a NanoDrop 2000 device (Thermo Fisher Scientific, USA). For the reverse transcription reaction, 500 ng of total RNA was used with the High-Capacity cDNA Reverse Transcription Kit (Roche, Germany). An expression analysis of the CD38 (Hs01120071_m1), OXTR (Hs00168573_m1), AVPR1A (Hs00176122_m1), and LNPEP (sonda Hs00893646_m1) genes was performed using TaqMan Gene Expression Assays, along with the reference gene GAPDH (Hs03929097_g1), and TaqMan Gene Expression Master Mix (Applied Biosystems, USA). Two replicates of the reaction for 50 ng of cDNA were carried out using a LightCycler480 II real-time thermal cycler from Roche, Germany. The expression results were analyzed using GenEx ver6 (MultiD Analyses AB, Sweden). Analyses of raw data were performed using the ΔCt method or relative expression as follows: raw data were normalized to technical repeats, to the amount of cDNA, and finally to the reference gene GAPDH. In the case of the analysis of ΔΔCt, i.e., the comparative method, an additional step to the calibrator was normalization.

### 2.5. Statistical Analysis

The statistical analysis was performed using StatSoft Statistica version 13 and JASP. The assumed level of statistical significance equaled α = 0.05. The assessment of the normality of the distribution was carried out using the Kolmogorov–Smirnov test. The analysis of the expression levels of the studied genes was carried out using the ΔΔCt. method. For the statistical analyses of the questionnaire results for gene expression levels, ΔCt. values were used. When comparing expression levels between subgroups within the study population, the following formula for the normalized value of the relative level of expression of the gene under study was used:

R = 2^−ΔΔCt^

where R = 1 means that the levels of expression between the analyzed groups are equal.

## 3. Results

**Figure 1:**
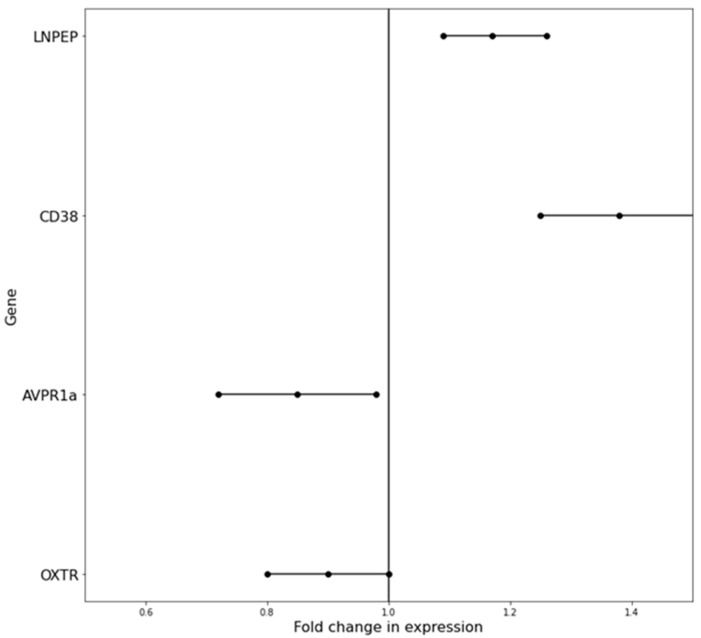
Fold change in expression R = log(ΔΔCt) with 95% confidence intervals for each of the studied genes.

Initially, the fold expression change between the study and control groups for each of the genes was analyzed. The obtained results are presented in Figure 1. In the case of the *LNPEP* and *CD38* genes, significantly higher levels of expression were observed in the group of people with ASD than in the neurotypical group—R_LNPEP_ = 1.17 (95% CI: 1.09–1.26) and R_CD38_ = 1.38 (95%CI: 1.25–1.51), respectively. In the case of the *AVPR1a* gene, the level of expression in the study group was slightly lower than in the control, with R_AVPR1a_ = 0.85 (95%CI: 0.72–0.98), while for the *OXTR* gene, the fold expression change obtained was similar between the two groups, with R_OXTR_ = 0.90 (95%CI: 0.8–1.01). Following this, a gender-by-grade analysis was performed, and it was observed that there was no significant difference in the *AVPR1a* and *OXTR* gene expression levels between the neurotypical and neuroatypical subjects in either the women nor men. Interestingly, fold expression change values in the male group increased from the non-grouped analysis for the *LNPEP* and *CD38* genes to R_LNPEP_ = 1.24 (95% CI: 1.13–1.36) and R_CD38_ = 1.44 (95%CI: 1.26–1.62), respectively.

**Figure 2:**
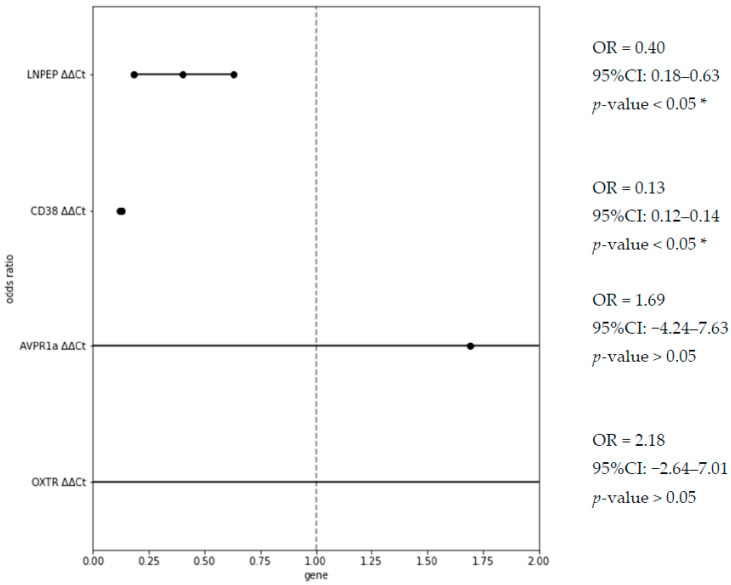
Odds ratio of belonging to the control group with increase in the expression level of the studied genes. * designates statistically significant odds ratio value.

A logistic regression analysis with a calculation of the odds ratio and 95%CI was also performed for the chance of belonging to the control (neurotypical) group depending on the level of expression of the analyzed genes. The obtained results are presented in Figure 2. The χ^2^ statistic equaled 15.97, and the *p*-value equaled 0.006.

Furthermore, an analysis of the Pearson correlation between the expression levels of the studied genes and the results of the utilized questionnaires was performed. In terms of expression levels in the study group, it was observed that the increase in *LNPEP* gene expression was associated with a statistically significant increase in the expression of other genes equal to r_OXTR_ = 0.29, r_AVPR1a_ = 0.31, and r_CD38_ = 0.56 and *p* < 0.05. A similar effect was not observed in the control group for the *OXTR* and *AVPR1a* genes but was present for the *CD38* gene (r_CD38_ = 0.48, with *p* < 0.05).

**Figure 3:**
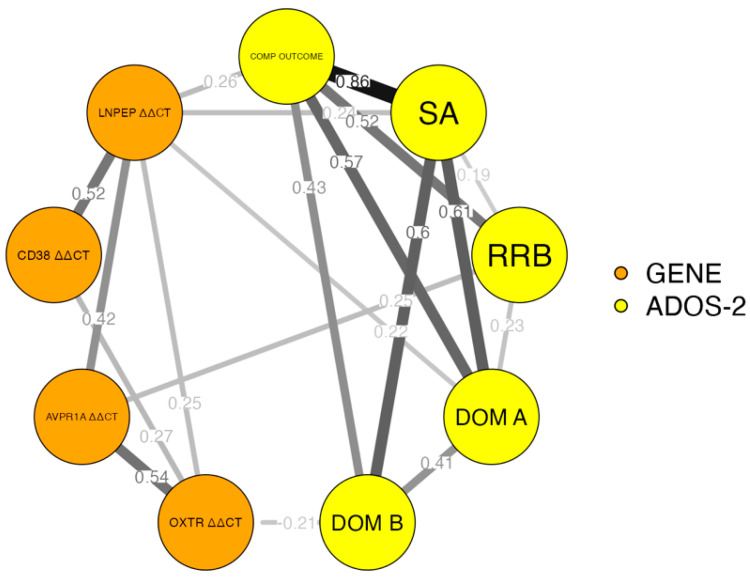
Network analysis of correlations between expression levels of analyzed genes and clinical presentation of ASD in ADOS-2 subscales (only significant edges are presented).

A network analysis based on Pearson’s correlation of expression levels of the studied genes with the ADOS-2 and BINET IQ subscales was also performed. The obtained results are presented in Figure 3. Significant correlations were demonstrated for the relationship between the *LNPEP* expression level and ADOS-2 comparative score (r = 0.26; *p* < 0.05), social affect subscale (r = 0.24; *p* < 0.05), and ADOS-2 communication and language disorder domain (r = 0.22; *p* < 0.05). A statistically significant correlation was also detected between the level of expression of *AVPR1a* and the subscale of restricted and repetitive behaviors, ADOS-2 (r = 0.25; *p* < 0.05). In addition, the Pearson correlation analysis also detected a statistically significant relationship between the BINET IQ score and the level of expression of the *CD38* gene (r = −0.409; *p* = 0.013). The correlation matrix for all analyzed parameters is presented in Table 1.

**Figure 4:**
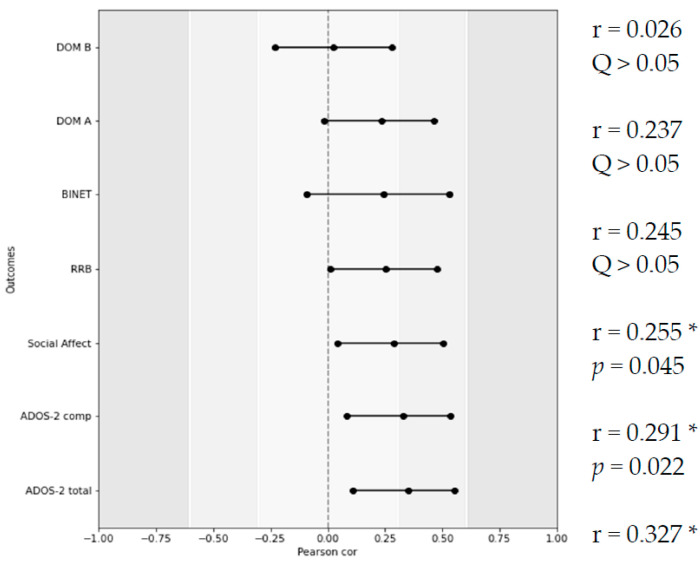
R-values of Pearson’s correlation between LNPEP/CD38 expression ratio, ADOS-2 subscales, and BINET IQ; DOM A—language and communication disorders; DOM B—disorder of social reciprocity; RRB—restrictive and repetitive behaviors. * designates statistically significant rho value.

Due to the opposite biological effect and moderate correlation between the expression levels of the *LNPEP* gene (breakdown of vasopressin and oxytocin) and *CD38* (secretion of vasopressin and oxytocin), it was hypothesized that a simultaneous increase in the expression of both genes may mitigate their impact on the examined parameters. Consequently, the ratio of ΔΔCt values for both genes was calculated, and the obtained coefficient was correlated with the ADOS-2 subscales and BINET IQ. Outcomes are presented in Figure 4.

None of the analyzed correlations obtained statistical significance in the analysis within the control group. The combined analysis of the groups yielded numerous significant correlations, but they are most likely the result of a statistically significant difference between the expressions in both groups and a significant difference in the results of the analyzed questionnaires.

## 4. Discussion

Oxytocin and vasopressin are neuropeptides that are often associated with autism spectrum disorders [5], although data linking their concentrations with the clinical picture of ASD are heterogeneous and often contradictory. This is most likely due to the nature of this nosological unit and its multifactorial background. The available literature indicates that ASD is determined by numerous polymorphisms in a wide range of genes. None of them play a key role in pathogenesis but are rather responsible for some small part of the clinical picture. Therefore, each patient might have a different array of mutations that leads to the development of a wide variety of symptoms that are considered “autistic”. Therefore, it should be expected that in the studies searching for genetic correlates of the observed symptoms, statistical parameters will be weak or moderate due to the inevitable heterogeneity of genotypes in the study group. Furthermore, the effects of oxytocin and/or vasopressin deficiency may also occur subsequently to defects and/or insufficiency of other proteins that play a role in their metabolism and function.

The half-lives of both of these neuropeptides are about 20 min within the cerebrospinal fluid [18]. The protein responsible for their breakdown within the central nervous system is LNPEP, but due to its preference for oxytocin, it is considered primarily an oxytocinase. Of course, in addition to this, it also shows activity against vasopressin. Despite the fact that this protein appears to be an interesting candidate in ASD substrate studies, at the moment, there are no studies within the PubMed database analyzing the impact of polymorphisms/expression levels in the *LNPEP* gene on the risk and clinical picture of ASD. We are aware of only one publication in the current literature linking LNPEP with a human disorder, and this was a study on the role of this oxytocinase in the development of septic shock. The authors, in addition to the effect on patient survival, confirmed that this protein actually has an effect on vasopressin concentrations as well [19]. Thus, our study seems to be the first analysis of its kind on the hypothetical role of *LNPEP* in ASD. Results from our study revealed its significantly higher expression in the study group in comparison with the neurotypical population. Furthermore, an increase in *LNPEP* expression was significantly associated with an increased risk of ASD. In terms of the clinical picture, oxytocinase was associated in a weak but statistically significant way with the ADOS-2 subscale of social affect and the domain of communication and social relations.

Another protein that plays a key role in the metabolism of oxytocin and vasopressin is the CD38 transmembrane glycoprotein, which plays a key role in their secretion [20]. There are several studies in the literature linking deviations of this protein to social competence in humans; however, to the best of our knowledge, there are currently only seven studies focusing specifically on the population of people with ASD—four studies analyzing solely polymorphisms, two papers analyzing both expression levels and polymorphisms, and one analyzing post mortem expression levels in cerebral tissues. The first is the Munesue et al. (2010) [10] analysis, where a statistically significant association was found between the “C” allele of the rs3796863 polymorphism and the risk of developing ASD in the American population. Interestingly, the authors did not observe such an association in the Japanese population. This study was followed by Higashida et al. (2013) [21], who demonstrated a significant association of the polymorphism rs1800561 (4693C > T) with ASD. It was also observed that it had a significant biological effect, causing a structural change in the CD38 protein (arginine substitution with tryptophan in codon 140) and being associated with lower oxytocin levels. The Hovey et al. (2014) [22] study showed a link between the “C” allele of SNP rs6449182 in *CD38* and the diagnosis of ASD, but the authors pointed to the inconclusivity of the results. A study by Lerer et al. (2010) [11] analyzed polymorphisms in the *CD38* gene in low-functioning people on the autism spectrum with cooccurring intellectual disability (IQ < 70). It is also one of two papers available in the literature that also analyze *CD38* expression levels. The authors analyzed haplotypes involving several polymorphisms and showed a statistically significant association between the “C” allele rs3796863 and the risk of ASD. Furthermore, they showed that this polymorphism is also related to the decrease in the level of expression of the *CD38* gene. Similarly, in the Riebold et al. [23] study, reduced levels of CD38 protein expression were observed in ASD patients, and concomitantly, they were associated with the Vineland Adaptive Behavior Scale outcome and the genotype of the polymorphism rs6449182 (C > G variation). Meanwhile, the opposite results were presented by Thanseem et al. (2012) [12], where the authors showed an increase in the *CD38* gene expression in cerebral tissues taken post mortem from 8 people diagnosed with ASD in comparison with tissues taken from a group of 13 neurotypical subjects. The results of the study by Thanseem et al. coincide with the data obtained in the course of the present study, where the diagnosis of ASD was associated with an approximately 38% increase in the expression of the *CD38* gene. Moreover, this result also translated into a higher risk of diagnosis linked to the increase in gene expression in a logistic regression analysis. What is the explanation for this type of heterogeneity in the results? Again, it should be noted that the etiology of ASD is diverse, and it is possible that some patients may present symptoms of lowering oxytocin levels secondary to the defect of the CD38 protein, and some may present them secondary to the increased activity of LNPEP oxytocinase. It is noteworthy that in the studied population, the increase in the level of LNPEP expression was moderately correlated with the level of *CD38* expression. This may suggest that an increase in *CD38* expression is a compensatory response to greater LNPEP expression. In the context of this observation, it was decided to carry out an analysis using the *LNPEP* to *CD38* gene expression ratio in order to control the obtained results for the protective effect of the CD38 protein. In fact, the analysis conducted in this way showed that the obtained coefficient correlated moderately with the result of the total ADOS-2 study and was also poorly correlated with the domains of social affect and repetitive and stereotyped activities. Therefore, it can be hypothesized that deviations in the level of expression of the *LNPEP* and *CD38* genes are significantly involved in shaping the risk and clinical picture of ASD.

This effect is, of course, indirect. The balance between the expression levels of the *CD38* gene and the *LNPEP* gene has a significant effect on vasopressin and oxytocin concentrations, which, in turn, is a hypothetical direct cause of the observed deficits. Obviously, the obtained correlation coefficients are low and are on the border of a weak/moderate correlation, but it should be remembered that according to the adopted paradigm, deficits within the oxytocinergic and vasopresynergic systems are to only be an element of the ASD substrate responsible specifically for disorders within social cognition. This hypothesis is reflected in studies on the concentrations of both of these neurohormones in the population of people with ASD [5] and also in the observed effectiveness of targeted pharmacotherapy for these neuropeptides in some of the studies available in the literature. One of the directions of research on pharmacological interventions in ASD that arouses more interest in the literature is the use of intranasal oxytocin. This interest is subsequent to the optimistic results of studies on the neurotypical population, where improvements in competence and social functioning were observed. Positive results on the efficacy of intranasal oxytocin were presented by, inter alia, Parker et al. (2017) [24], pointing to the effectiveness of such intervention in diminishing disorders of social functioning in children with ASD, as well as Seeley et al. [25], showing that intranasal oxytocin significantly modulates resting state functional connectivity. However, a literature review and meta-analysis of studies on this intervention conducted by Wang et al. in 2019 [26] showed that, compared to a placebo, oxytocin use had no statistically significant effects on ASD axial symptoms (social functioning: SMD = 0.03; 95%CI: −0.19 to 0.25; *p* = 0.7; repetitive and stereotyped patterns of functioning and activity: SMD = 0.01; 95% CI: −0.26 to 0.27; *p* = 0.9); however, the analyzed publications presented a high heterogeneity of results. In the case of vasopressin, two publications on this topic are currently available in the PubMed database. Bolognani et al. (2019) [27] conducted an analysis on a group of 223 men diagnosed with ASD using balovaptane at a dose of 4–10 mg, obtaining a significant, higher than placebo, improvement after 12 weeks of use in communication scales and social skills of the Vinland-II Adaptive Behavior Scale. Similarly, in 2017, Umbricht et al. [28] showed improvements in social cognition skills in a group of 19 people with ASD using the strong vasopressin receptor antagonist RG7713.

Of course, at this point, an interesting direction for further research would be a combined analysis of *LNPEP* and *CD38* gene expression levels together with oxytocin/vasopressin concentrations aimed at verifying the presented relationships. However, at the moment, according to our knowledge, there are no such analyses available in the literature.

Other genes that play an important role in the functioning of the oxytocinergic and vasopresynergic systems are the *OXTR* and *AVPR1a* genes. The *OXTR* gene, in particular, has aroused great interest among scientists in recent years. Several studies are available in the literature analyzing the relationship between single nucleotide polymorphisms and the risk and clinical picture of ASD for both receptors’ genes [9]. It should be noted, however, that polymorphisms that are associated with the occurrence of ASD are located in non-coding areas, and therefore, their biological effect will be primarily, if at all, related to the regulation of the expression of these genes. Therefore, the genotype of the studied polymorphisms should translate into changes in the level of their expression. To our knowledge, there are two studies in the available literature examining the relationship between *OXTR* gene expression and ASD diagnosis. The first, by Frehner et al. (2022) [29], was conducted post mortem on the brain tissues of neuroatypical and neurotypical subjects and showed no significant differences in the expression of this gene between groups. The second, by Voinsky et al. (2019) [30], also showed no statistically significant associations between the level of expression of the *OXTR* gene and the risk of developing ASD. Interestingly, however, the latter study showed a significant correlation of *OXTR* gene expression with symptoms of social functioning disorders measured by the Aberrant Behavior Checklist (ABC), the Vineland Adaptive Behavior Scale (VABS), and the Social Responsiveness Scale (SRS). In the present study, *OXTR* gene expression also had no significant effect on the risk of ASD diagnosis and was not associated with its clinical picture. For the *AVPR1a* gene, there was only one study available in the literature by Voinsky et al. [30], and no significant correlation between its expression and ASD diagnosis was found. However, the authors showed an association with the clinical picture of ASD as part of the Aberrant Behavior Checklist (ABC). Interestingly, in the present study, similarly to the *OXTR* gene, *AVPR1a* expression levels did not present a significant association with the risk of ASD diagnosis in a logistic regression analysis. Nevertheless, it was about 20% lower in the neuroatypical group than in the neurotypical group and showed a weak, positive correlation with the domain of stereotyped and repetitive patterns of interest and activity. This result is consistent with the accepted hypotheses, as it was the reduction in *AVPR1a* expression that was expected in the study group. Interestingly, however, contrary to our expectations, this deviation did not translate into either the actual risk of developing ASD compared to the control group or the clinical picture of social cognition deficits.

Discussing the obtained results in the context of polymorphisms in the relevant genes analyzed in the literature, the obtained expression levels may reflect their regulatory impact—most of the polymorphisms studied in the literature are located in the intron area without affecting the structure of encoded proteins. However, in order to link expression levels to elements of the clinical picture, post-translational factors that affect the function of proteins themselves, regardless of their expression levels, should be considered. In the case of the LNPEP and CD38 proteins, glycosylation is the main mechanism of modification, which affects the stability of proteins, cellular localization, and, above all, the level of enzymatic activity. In the case of OXTR and AVPR1a, the essential post-translational modification process is phosphorylation, which not only affects receptor internalization but also modifies intracellular transmission in response to a ligand. Additionally, alternative splicing of AVPR1a can yield receptor variants with different pharmacological properties. These post-translational modifications and regulatory mechanisms play pivotal roles in fine-tuning the functions of these genes’ protein products. They affect aspects such as enzymatic activity, receptor responsiveness, and protein stability, all of which can ultimately impact their biological functions in processes like hormone signaling and enzymatic activity. Therefore, considering these post-translational modifications is crucial for a comprehensive understanding of the roles of LNPEP, CD38, OXTR, and AVPR1a in biological systems. The present study did not analyze these mechanisms or directly assess the level of activity of, for example, LNPEP. For this reason, great caution should be exercised when interpreting the presented results in the context of the biological effect, and research on this issue is necessary in the future.

The main limitation of the presented study is the disproportion between the size of the study and the control group and their generally limited size. However, it should be noted that at the design stage of the study, a statistical power analysis was performed for the correlation of expression levels with the ADOS-2 score and the differences in the student’s *t*-test for both groups in the ADOS-2 result, and the obtained statistical power parameters were *t*-student = 0.982 and cor = 0.979, respectively (for r = 0.4). These results indicate that the number of patients assumed in the study should provide sufficient statistical power to accept the obtained results as reliable. Another issue is the statistically significant age difference observed between the study and control groups. However, according to the characteristics of the tests used in the study, including primarily the ADOS-2 protocol, this difference should not significantly affect the results obtained. Finally, as already mentioned, for a full interpretation of the presented results in terms of the biological effect of the observed dependencies, it would be necessary to simultaneously determine the level of oxytocin and vasopressin, i.e., in peripheral blood. However, this was technically impossible at this stage of the investigation.

Summing up, the results obtained in the presented study indicate that in the field of oxytocinergic and vasopresynergic systems, the balance between the levels of expression of the *CD38* gene and the *LNPEP* gene plays a key role in the risk and clinical presentation of ASD. Imbalance in their expression hypothetically translates into disruption of oxytocin and vasopressin concentrations and, thus, indirectly shapes the clinical picture of social deficits in this group of people. At the same time, the most frequently studied genes—*AVPR1a* and *OXTR*—seem to be at best of marginal importance for the risk of ASD. This is an interesting result in the context of the fact that polymorphisms commonly analyzed in the literature are generally located in areas responsible for the regulation of gene expression. Therefore, if they were actually to have a biological effect, they should impact the level of expression of these genes. Finally, in the case of the *AVPR1a* gene, despite the lack of a significant relationship between the risk of ASD and its level of expression, a significant reduction (by about 20%) in the level of its expression between groups was observed. This may indicate that the vasopressin receptor is in a limited way (e.g., in some patients) associated with the diagnosis of ASD; however, its effect is heterogeneous among the subjects, which made it impossible to obtain a statistically significant odds ratio.

## 5. Conclusions

1. The balance between *CD38* and *LNPEP* gene expression seems to significantly impact ASD risk and clinical presentation in the analyzed population;
2. The majority of studies on the *OXTR* gene correlate ASD risk and presentation with polymorphisms located in regulatory areas. The study expression levels of *OXTR* seem to be at best of marginal importance and, therefore, suggest that widely studied polymorphisms may have no significant biological effect;
3. *AVPR1a* seems to be in a limited way (e.g., in some patients) associated with the diagnosis of ASD; however, its effect may be heterogeneous among the subjects, which makes it impossible to obtain a statistically significant odds ratio for the risk of ASD.

## Figures and Tables

**Table 1:** Correlation matrix for all analyzed parameters; Spearman’s rho value on the left side; *p*-value on the right side. CS = cumulative scale; SA = social affect; RRB = restrictive and repetitive behaviors; DA = domain A; DB = domain B.

	LNPEP ΔΔCt	CD38 ΔΔCt	AVPR1a ΔΔCt	OXTR ΔΔCt	ADOS-2 CS	ADOS-2 SA	ADOS-2 RRB	ADOS-2 DA	ADOS-2 DB
LNPEP ΔΔCt	X	0.0001	0.0023	0.0042	0.0001	0.00001	*p* > 0.05	0.006	*p* > 0.05
CD38 ΔΔCt	0.52	X	*p* > 0.05	0.0032	*p* > 0.05	*p* > 0.05	*p* > 0.05	*p* > 0.05	*p* > 0.05
AVPR1a ΔΔCt	0.42	−0.01	X	0.023	*p* > 0.05	*p* > 0.05	0.00695	*p* > 0.05	*p* > 0.05
OXTR ΔΔCt	0.25	0.27	0.54	X	*p* > 0.05	*p* > 0.05	*p* > 0.05	*p* > 0.05	*p* > 0.05
ADOS-2 CS	0.26	0.03	0.08	0.11	X	*p* < 0.0001	*p* < 0.0001	*p* < 0.0001	*p* < 0.0001
ADOS-2 SA	0.24	0.08	−0.1	0.09	0.86	X	0.005	0.00042	0.00012
ADOS-2 RRB	0.04	−0.04	0.25	0.01	0.52	0.19	X	0.026	*p* > 0.05
ADOS-2 DA	0.22	0.01	−0.02	−0.03	0.57	0.6	0.23	X	*p* < 0.0001
ADOS-2 DB	0.09	−0.02	−0.04	0.21	0.43	0.61	0.09	0.41	X

## Data Availability

The datasets used and/or analyzed during the current study are available from the corresponding author on reasonable request.
